# Seeing Is Believing: How Does the Surface of Silver Nanocubes from a Polyol Synthesis Change during Sample Collection, Washing, and Redispersion

**DOI:** 10.1021/acs.langmuir.5c02610

**Published:** 2025-07-22

**Authors:** Qijia Huang, Younan Xia

**Affiliations:** † School of Chemistry and Biochemistry, 1372 Georgia Institute of Technology, Atlanta, Georgia 30332, United States; ‡ The Wallace H. Coulter Department of Biomedical Engineering, Georgia Institute of Technology and Emory University, Atlanta, Georgia 30332, United States

## Abstract

While the synthesis of Ag nanocubes has been extensively studied, sample preparation (including collection, washing, and redispersion) after the synthesis has received far less attention. Herein, we leverage the unique capability of surface-enhanced Raman scattering to investigate how the solvent used for sample preparation affects the surface chemistry of Ag nanocubes. Our findings reveal that the use of an appropriate solvent for sample preparation plays a vital role in preserving the cubic shape. Crushing the reaction mixture with acetone before centrifugation greatly improves collection efficiency by inducing reversible aggregation among the particles. It also promotes the coadsorption of the carbonyl group from acetone and Cl^–^ ions on the Ag surface to suppress oxidative etching and thereby help preserve the cubic shape. Subsequent washing of the collected nanocubes with water or ethanol enables effective redispersion while facilitating the desorption of Cl^–^ ions and the adsorption of the carbonyl group from poly­(vinylpyrrolidone). Collectively, these results underscore the importance of processing conditions after a colloidal synthesis in preserving the desired properties of Ag nanocubes for an array of applications.

## Graphical abstract

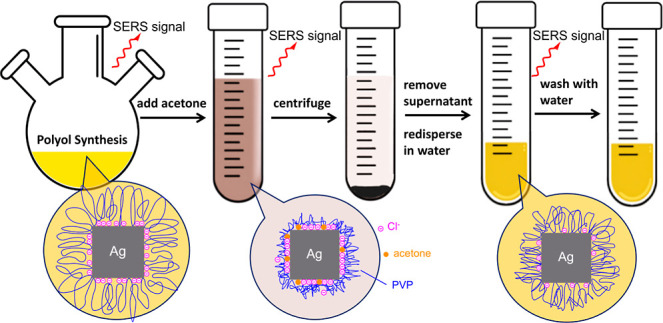

## Introduction

Since the report of a successful synthesis based upon the polyol method in 2002,^1^ Ag nanocubes have received considerable interest for a range of applications. For example, owing to their sharp corners and edges, Ag nanocubes exhibit unique optical properties related to localized surface plasmon resonance (LSPR)^2,3^ for applications in optical sensing and surface-enhanced Raman scattering (SERS).^4–7^ Additionally, Ag nanocubes are enclosed by {100} facets, giving them high catalytic selectivity toward ethylene epoxidation,^8^ as well as attractive features for fundamental studies such as crystal growth,^9,10^ surface adsorption/desorption,^11^ and colloidal assembly.^12,13^ Over the past two decades, polyol synthesis has evolved as the method of choice for Ag nanocube synthesis owing to its excellent reproducibility and tunability.^1,14–19^ In a typical synthesis, a Ag­(I) precursor is reduced in ethylene glycol (EG), which serves as both a solvent and a precursor to glycolaldehyde, the actual reducing agent. A number of chemical species have been introduced into the original protocol to help enhance the robustness and tunability of the synthesis. In the most recent version of the protocol,^18^ CF_3_COOAg serves as a more reliable Ag­(I) precursor than AgNO_3_ ^15^ due to its ability to prevent the introduction of uncertainties arising from the formation of HNO_3_ and possible decomposition of NO_3_^–^ at an elevated temperature; NaSH is introduced to shorten the reaction time by accelerating nucleation;^20^ and HCl is used to promote oxidative etching of unwanted twinned seeds while acting as a capping agent coadsorbed with poly­(vinylpyrrolidone) (PVP) on the {100} facets.^21,22^ Owning to the efforts from various groups, the roles played by most of these chemical species in controlling the formation of Ag nanocubes have been mostly elucidated and understood.

Relative to the extensive efforts on synthesis, there is essentially no study on how the sample preparation after a synthesis would affect the surface properties of the Ag nanocubes. In general, sample collection, washing, and redispersion are treated as routine procedures without considering their potential impact on the surface of Ag nanocubes or other types of colloidal nanocrystals. Given the high sensitivity of Ag surface to ligand exchange and oxidative etching under ambient atmosphere,^23^ sample preparation may result in surface ligand exchange, particle aggregation, and/or truncation to their sharp corners and edges.^24^ Such a change inevitably compromises the optical and catalytic properties of Ag nanocubes. There is an urgent need to address this issue by developing analytical methods capable of revealing the surface changes while the nanocrystals are washed with and then dispersed in different solvents.

Extensive studies have established that SERS is a powerful technique for probing changes to the surface of plasmonic metal nanocrystals (such as those made of Au, Ag or Cu) while they are suspended in a liquid phase.^25–27^ In this study, we seek to elucidate the changes to the surface of Ag nanocubes during sample preparation using SERS, and understand how different solvents used for collection, washing, and dispersion affect their surface chemistry and shape stability. We found that the Ag nanocubes synthesized using the HCl-mediated polyol method underwent corner and edge truncations within several hours if they were not collected, washed, and redispersed after the synthesis. Simply diluting the original reaction mixture with EG was unable to prevent the shape change. In contrast, if the solution was diluted with acetone, water, or ethanol, the Ag nanocubes could retain the cubic shape even after storage under the ambient atmosphere for 10 h. We further analyzed the surface changes during sample collection and washing by SERS. Upon the addition of acetone, we observed aggregation of nanocubes and a redshift in the stretching mode initially attributed to Ag–Cl, which could be ascribed to the coupling of vibrational modes of Ag–Cl and Ag–O (involving the carbonyl group from acetone). Continued washing with water led to a further redshift of this peak due to the additional contribution from the carbonyl group of PVP. We also performed SERS measurements on the samples diluted to the same extent with EG, water, acetone, and ethanol and our results suggested that acetone promoted coadsorption of the carbonyl group from acetone and Cl^–^ ions, whereas water and ethanol mainly facilitated the adsorption of the carbonyl group from PVP at the unoccupied surface sites. Both mechanisms effectively inhibited oxidative etching, helping preserve the sharp corners and edges on the nanocubes.

## Materials and Methods

### Chemicals and Materials

Poly­(vinylpyrrolidone) with an average molecular weight of 55,000 (PVP-55k, lot no. MKCD1968), silver trifluoroacetate (CF_3_COOAg, ≥99.99%, trace metal basis, lot no, MKBZ0931 V), sodium hydrosulfide hydrate (NaHS·*x*H_2_O, lot no. SHBP0761), and aqueous hydrochloric acid (HCl, 37%, lot no. 24006167) were all ordered from Sigma-Aldrich. Ethylene glycol (HOCH_2_CH_2_OH, EG, lot no. 0000160068) was purchased from J. T. Baker. Acetone (HPLC grade, 99.5+%) was obtained from Alfa Aesar. All the chemicals were used as received. Deionized (DI) water with a resistivity of 18.2 MΩ·cm at room temperature was used in all experiments.

### Synthesis of Ag Nanocubes

We followed a published protocol to synthesize the Ag nanocubes.^18^ In a standard synthesis, 0.24 mL of NaSH (3 mM in EG), 2.0 mL of HCl (3 mM in EG), 5.0 mL of PVP-55k (20 mg/mL in EG), and 1.6 mL of CF_3_COOAg (282 mM in EG) were sequentially introduced into 20 mL of preheated EG (150 °C) contained in a 100 mL round-bottom flask. After the introduction of CF_3_COOAg, the reaction progress was monitored in real time by tracking the position of the main LSPR peak using a UV–vis spectrometer. Specifically, a few drops of the reaction solution were withdrawn from the flask using a glass pipet and diluted with water in a cuvette, followed by the collection of its extinction spectrum. Once the main LSPR peak reached 428 nm, the reaction was immediately quenched by immersing the flask in an ice bath. During the synthesis, the flask was capped with glass stoppers except for adding reagents or collecting samples for UV–vis monitoring. The solid product was crushed out using acetone, washed with water twice, and then dispersed in water for TEM sample preparation. A more detailed description of the protocol can be found in a prior publication.

### Raman and SERS Measurements

We used Si(100) as a standard sample to calibrate the Raman spectrometer before Raman and SERS measurements. In a typical process, we transferred the aliquot into a cell made of poly­(dimethylsiloxane) (PDMS), covered with a glass coverslip to prevent solvent evaporation. The surface of the coverslip was also used as a reference point to position the focal plane 40 μm into the liquid sample during the measurements. Spectra were collected in the extended mode at an excitation wavelength of 532 nm, together with a 100× objective lens, a laser power at 25 mW, and a collection time of 10 s. An aliquot of 25 μL was withdrawn from each diluted sample for Raman and SERS measurements.

### SERS Measurements during the Sample Preparation after Synthesis

In a typical polyol synthesis of Ag nanocubes, the reaction solution was quenched in an ice bath, followed by the addition of acetone (three times in volume) and then centrifugation to crush out and collect the solid products. After that, the sample was washed twice with water and finally redispersed in water. After each centrifugation step, ultrasonic treatment was used to redisperse the Ag nanocubes in the solvent. An aliquot of 25 μL was withdrawn after each step and used for SERS measurement.

### SERS Measurements of Ag Nanocubes Dispersed in EG-Acetone Binary Mixtures with Different Ratios

We collect the SERS spectra of Ag nanocubes dispersed in the original reaction solution or in EG-acetone binary mixtures with different ratios. The ratios of EG to acetone were 2:1, 1:1, 1:2, and 1:3, respectively. All these samples for SERS measurements were prepared from the original reaction solution by dilution at a fixed dilution factor of 4 with EG or acetone. For example, to prepare a sample with an EG/acetone ratio of 2:1 under this dilution scheme, 100 μL of EG and 80 μL of acetone were introduced into 60 μL of the original EG-based reaction solution simultaneously.

### SERS Measurements of Original Reaction Solution of Ag Nanocubes Diluted with Different Solvents

We collected SERS spectra of the Ag nanocubes after the reaction mixture was diluted by a factor of 4 with EG, water, acetone, and ethanol, respectively. The samples were kept for 40 min prior to SERS measurement.

### Instrumentation and Characterizations

We used a centrifuge (Eppendorf 5430) to collect and wash all solid products. A Cary 50 spectrometer (Agilent Technologies, Santa Clara, CA) was used to record the UV–vis spectra. Transmission electron microscopy (TEM) images were taken using a Hitachi HT7700 microscope (Japan) operated at 120 kV. The Raman and SERS spectra were recorded using a Renishaw inVia Raman Spectrometer (Wotton-under-Edge, U.K.) integrated with a Leica microscope (Wetzlar, Germany).

## Results and Discussion

Figure S1 shows a TEM image of the Ag nanocubes synthesized using the HCl-mediated polyol protocol.^18^ They had an average edge length of 32.2 ± 5.3 nm and their surface was supposed to be passivated by both Cl^–^ ions and PVP. Without the collection, washing, and redispersion steps, it was difficult to preserve the sharp corners and edges on the Ag nanocubes once the reaction had been quenched in an ice bath. Figure S2 shows UV–vis spectra of the Ag nanocubes after storage at room temperature and under ambient conditions in the original reaction solution without/with dilution with different solvents. When the Ag nanocubes were stored in the original reaction solution for 0.5 h, their UV–vis spectrum exhibited a primary extinction peak at 437 nm, together with a shoulder peak around 350 nm, indicating the presence of sharp corners and edges.^3^ After storage for 10 h, the primary peak blue-shifted to 414 nm while the shoulder peak disappeared. This blue shift indicated a reduction in particle size, whereas the disappearance of the shoulder peak could be attributed to the truncation of both the corners and edges for the formation of a more spherical shape. When the original reaction solution was diluted by a factor of 4 in volume with EG, the UV–vis spectrum showed similar changes when stored for 10 h. However, if the reaction solution was diluted with acetone, water, and ethanol, respectively, the shoulder peak still existed after 10 h of storage, indicating that the sharp corners and edges were preserved in all these three solvents. The slight shifts to the primary LSPR peak in these three solvents could be attributed to the change in refractive index for the dispersion medium, together with the possible changes in particle size.^28^ Additionally, the peak broadening observed for the sample diluted with acetone could be ascribed to particle aggregation as acetone is a known bad solvent for the PVP adsorbed on the surface of the nanocubes.^29^

We also conducted a control experiment by placing the original reaction solution under argon protection. This sample still exhibited a well-resolved shoulder peak at 350 nm after storage at room temperature for 10 h, suggesting that the corners and the edges were likely truncated through an oxidative etching process caused by the O_2_ dissolved in the EG. However, since O_2_ has the lowest solubility in EG compared to acetone, water, and ethanol,^30^ we hypothesized that these three solvents somehow interacted favorably with the surface caping ligand (Cl^–^ and PVP) on Ag nanocubes to prevent oxidative etching from occurring. For all the control groups mentioned above, after the collection of UV–vis spectra, the nanocubes were collected and prepared for TEM imaging using the standard protocol: acetone was added to induce precipitation, followed by two washes with water and final redispersion in water. The TEM image shown in Figure S3 confirmed that without the introduction of acetone, water, and ethanol, respectively, or protection by argon, respectively, the Ag nanocubes tended to be truncated in the EG-based reaction solution. Interestingly, Ag nanocubes in Figure S3c seem to be more truncated compared to those in Figure S3b, but the UV–vis spectrum in Figure S2c shows a more pronounced shoulder peak compared to Figure S2b. This difference is subtle but may be attributed to the size difference between the two samples. As reported in one study, even a small increase in edge length (e.g., from 23 to 28 nm) can lead to a noticeable enhancement in the shoulder peak despite minimal shifts in the main LSPR band.^17^ In our case, the main peak in Figure S2c is located at 418 nm, whereas that in Figure S2b appears at 414 nm, indicating that the sample in S2c likely contains slightly larger nanocubes. This minor size difference may be responsible for the enhanced shoulder feature observed. These observations suggest that using different solvents to collect and wash the solid products after a polyol synthesis plays a vital role in better passivating the surface of the Ag nanocubes to help preserve their sharp corners and edges. It is the intention of this work to understand the nature of such passivation using SERS.

In a typical polyol synthesis of Ag nanocubes, the reaction solution was quenched in an ice bath, followed by the addition of acetone (three times in volume) and then centrifugation to crush out and collect the solid products. Afterward, the sample was washed twice with water and finally redispersed in water. To elucidate the possible changes to their surface, we leveraged the finger-printing capability of SERS to analyze the Ag nanocubes at each step of sample preparation. As shown in Figure 1, the SERS spectrum of the nanocubes in the original reaction solution displayed a peak at 240 cm^–1^, which could be assigned to the stretching mode of AgCl (ν_Ag–Cl_) based upon our previous studies.^22^ The intensity of this peak increased significantly after the introduction of acetone, despite the decrease in particle concentration. The augmentation in peak intensity could be attributed to two factors: (i) the lower dielectric constant of acetone (ca. 20.7) relative to EG (ca. 41.2) at ambient temperature,^31,32^ which reduced the solubility of AgCl in the acetone–EG mixture and thereby increased the amount of AgCl deposited on the surface of the Ag nanocubes in the acetone–EG mixture and (ii) the aggregation of Ag nanocubes induced by acetone, which led to the creation of hot spots in the gaps between neighboring particles to amplify the SERS signal.^33^

**Figure 1:**
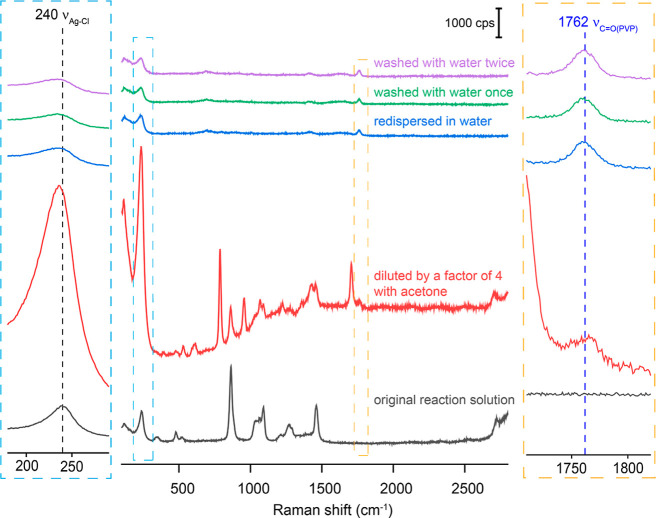
SERS spectra of the Ag nanocubes suspended in the original reaction solution; after dilution by a factor of 4 in volume with acetone; after centrifugation and then redispersion in water; after centrifugation and redispersion in water; after centrifugation, washing with water once, and then redispersion in water; and after centrifugation, washing with water twice, and then redispersion in water. The concentrations of Ag nanocubes in all aqueous samples were kept roughly the same as that of the original reaction solution.

The two factors noted above could also be used to account for the weakening of the Ag–Cl signal when the Ag nanocubes were further washed with water and finally redispersed in water. Water has a higher dielectric constant (ca. 78.4) than that of EG and acetone so that AgCl should be most soluble in water. Additionally, since water is a better solvent for PVP than acetone, the Ag nanocubes could be redispersed well in water, eliminating the formation of SERS hot spots due to aggregation.^29^ It is worth mentioning that the Ag–Cl peak red-shifted by approximately 3 cm^–1^ when acetone was introduced into the reaction solution, suggesting that the polarity of the solvent could affect the electron distribution in the Ag–Cl bond. Alternatively, this shift may result from the coadsorption of acetone on the surface of Ag nanocubes by binding through the oxygen atom in the carbonyl group.

It is worth noting that the ν_C=O_ peak of PVP was absent in the original reaction solution, as well as in the sample diluted with acetone, whereas it was visible at 1762 cm^–1^ in the sample redispersed in water. Based on our previous studies, the PVP loops would collapse since acetone and water are bad solvents. As such, more free carbonyl groups would get close to the Ag surface, increasing the intensity of the ν_C=O_ peak. However, the intensity of the ν_C=O_ peak in the SERS spectrum of the sample based on EG–acetone mixture was relatively weak although the PVP should collapse more than the case of water. This trend could be attributed to the following factors: (i) competitive adsorption of the carbonyl group of acetone on the surface of Ag nanocubes; (ii) the weakening of the SERS signal of the carbonyl group due to a thicker and denser AgCl solid on the surface of the nanocubes because of the poor solubility of AgCl in acetone, and (iii) more bonding sites on the nanocubes for the carbonyl group of PVP in an aqueous solution due to the dissolution of AgCl from the Ag surface.

To gain a better understanding of the surface of Ag nanocubes crushed out with acetone, we introduced increasing volumes of acetone into the original reaction solution to obtain EG–acetone binary mixtures with the ratio of EG to acetone at 2:1, 1:1, 1:2, and 1:3, respectively. Figure 2a shows a digital photograph of these samples, from which we could observe a color change from yellow to gray when the ratios of EG to acetone were 1:2 and 1:3, indicating aggregation of the nanocubes. Subsequently, we measured UV–vis and SERS spectra of Ag nanocubes dispersed in the original reaction solution or in EG–acetone binary mixtures with these ratios, using a fixed dilution factor. As shown in Figure 2b, a broad shoulder peak appeared at wavelengths beyond the major LSPR peak when the ratio of EG to acetone was set to 1:2, confirming that the nanocubes started to aggregate under this condition. At a ratio EG to acetone ratio of 1:3, the major LSPR peak was almost invisible, indicating severe aggregation and thus precipitation of the particles from the medium.

**Figure 2:**
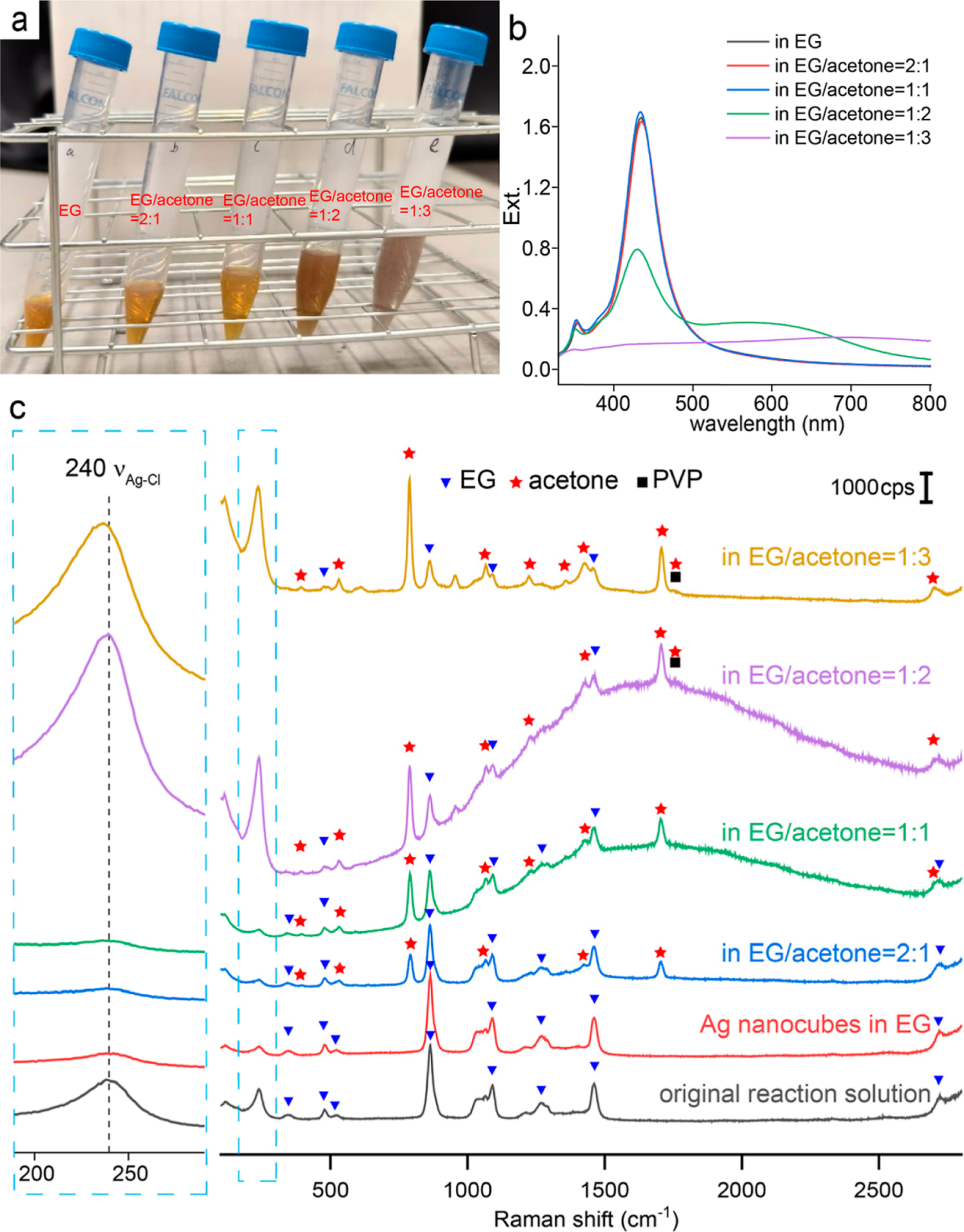
(a) Digital photograph, (b) UV–vis spectra, and (c) SERS spectra of Ag nanocubes dispersed in the original reaction solution or in EG–acetone binary mixtures with different ratios. The samples for UV–vis and SERS measurements were prepared from the original reaction solution by dilution with EG or acetone at a factor of 4.

Figure 2c shows SERS spectra recorded from the same samples. When comparing the SERS spectra of the samples without/with diluting the original reaction solution with EG by four folds, it is worth noting that the peak area of ν_Ag–Cl_ decreased to one-fourth of its original value, indicating that the SERS signal of Ag–Cl was directly proportional to the dilution factor. As the proportion of acetone used for dilution gradually increased, we observed that the intensities of the peaks corresponding to EG gradually decreased whereas the intensities of the peaks associated with acetone gradually increased. However, when the ratio of EG to acetone in the mixture was set to 2:1 or 1:1, the peak area and position of ν_Ag–Cl_ remained essentially the same. Although AgCl should have lower solubility in the mixture with increasing acetone proportion due to the lower dielectric constant of acetone relative to EG, the SERS signal of this peak did not increase, making it inconclusive whether more AgCl was deposited onto the surface of the nanocubes. However, when the ratio of EG to acetone was reduced to 1:2 or 1:3, the area of the peak increased by more than 16 times. Combined with the results from UV–vis measurements, we could infer that the enhancement of SERS signal should be mainly attributed to particle aggregation. Simultaneously, only in samples with EG to acetone ratios at 1:2 and 1:3, a weak peak at 1762 cm^–1^, corresponding to ν_C=O_, was observed.^27^ This peak confirmed the presence of carbonyl groups on the Ag surface. The carbonyl groups of both acetone and PVP could contribute to this signal. Interestingly, it was observed that ν_Ag–Cl_ shifted to about 235 cm^–1^ when the ratio of EG to acetone was set to 1:3. We argued that this shift was a result of mixing between the vibrational modes of ν_Ag–Cl_ and ν_Ag–O_ (due to the interaction between Ag and the oxygen of the adsorbed carbonyl). As the acetone concentration increased beyond a certain threshold, the competitive adsorption of carbonyl groups on the {110} and {111} facets became more pronounced. This increased adsorption led to the emergence of a composite vibrational mode involving both ν_Ag–Cl_ and ν_Ag–O_, resulting in the shift toward a lower wavenumber. It is worth noting that strong SERS background was observed in the samples involving EG to acetone ratios at 1:1 and 1:2, whereas the sample at a ratio of 1:3 exhibited a much weaker background. Although the exact origin of the SERS background is not yet fully understood, we speculate that it may arise from emission associated with molecular aggregates or clusters formed by the solvent molecules at specific ratios.^34^ Additionally, the ratio of SERS peak to background has been reported to be highly sensitive to the nanoscale morphology of the surface.^35^ In this study, the extent of aggregation of Ag nanocubes varied with solvent composition, which likely influenced the number and morphology of SERS hot spots. Further investigation, potentially involving in situ microscopic techniques to directly visualize aggregation and hotspot formation,^36^ is needed to clarify the mechanism behind this observation.

As shown in Figure 3a, when we redispersed the Ag nanocubes, which were crushed out with acetone and collected by centrifugation, in water while retaining 5% of the supernatant, a peak was observed at 237 cm^–1^. A subsequent washing with water further red-shifted this peak to 234 cm^–1^. The peaks corresponding to acetone confirmed the presence of acetone in the sample. In contrast, if more than 99% of the supernatant was removed before redispersing the sample in water, followed by washed with water once, the peak was red-shifted down to 231 cm^–1^ in both SERS spectra of the samples shown in Figure 3b. The shift could be attributed to two possible factors: (i) weakening of the Ag–Cl bond and (ii) an increased proportion of carbonyl oxygen adsorbed on the surface. In the former explanation, transferring Ag nanocubes from a less polar EG–acetone mixture to a highly polar aqueous environment enhanced the solvation of the adsorbed Cl^–^ ions. The stronger solvation weakened the Ag–Cl bond (i.e., reduction in the force constant), resulting in a lower vibrational frequency. If a considerable amount of acetone remained, the oxygen atoms of acetone could coordinate to the surface atoms on the Ag nanocubes in a manner similar to that of PVP. The presence of a significant amount of acetone on the Ag surface could create a local environment with a reduced effective polarity relative to an environment dominated by water. Thus, the weakening of the Ag–Cl bond was less significant than the case in pure water. The washing with water gradually reduced the proportion of acetone in the mixture, thereby decreasing the amount of acetone on the Ag surface. This, in turn, weakens the Ag–Cl bond, leading to a reduction in the vibrational frequency. For the latter explanation, due to the higher solubility of AgCl in water, the introduction of water significantly reduced the amount of AgCl on the Ag surface. As a result, the contribution to the SERS signal from the interaction between carbonyl oxygen and Ag increased, leading to a redshift for the peak. Additionally, as the acetone concentration decreased, the competitive adsorption of PVP carbonyl groups on the Ag surface became more pronounced, enhancing the intensity of the carbonyl peak at 1762 cm^–1^. This peak could be attributed to a combination of carbonyl groups directly adsorbed on the Ag surface and those associated with PVP molecules dispersed in the solvent while in proximity to the Ag surface.

**Figure 3:**
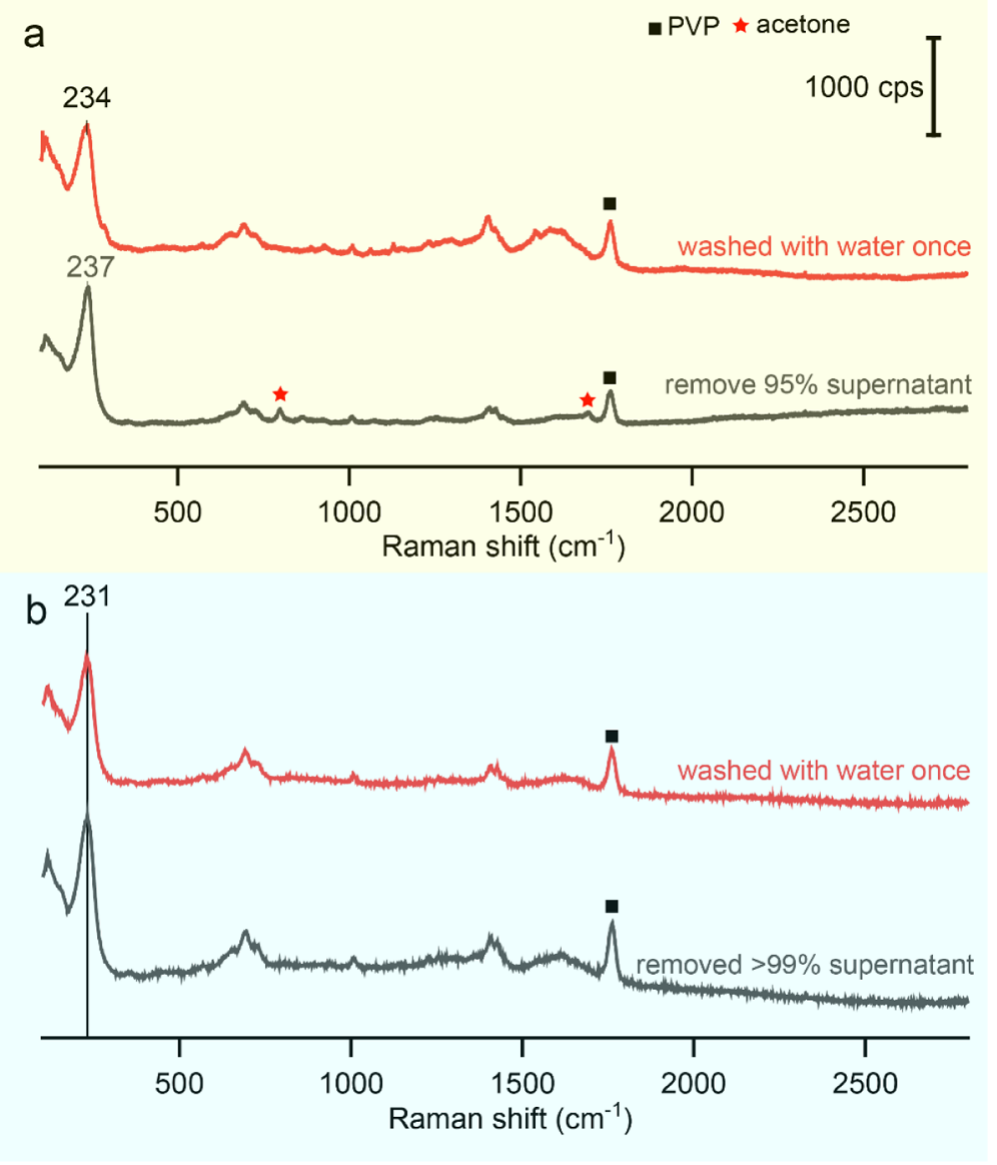
SERS spectra recorded from the Ag nanocubes collected by centrifugation after being crashed with acetone while removing (a) 95% and (b) >99% of the supernatant, respectively, followed by redispersion in water, with the sample washed with water once.

According to the above results, it is clear that the surface of the Ag nanocubes prepared using the HCl-mediated polyol method undergoes a range of changes during the collection and washing steps. As illustrated in Figure 4, the Ag nanocubes are well dispersed in the original reaction solution, with PVP extending well into the surrounding EG to form large loops. Additionally, a significant amount of Cl^–^ coadsorbs with PVP on the Ag surface. When acetone of three times in volume is added, the poor solubility of PVP in acetone would collapse the PVP loops, resulting in major reduction in the steric effect and thus particle aggregation. Meanwhile, the abundance of carbonyl group from acetone leads to its coadsorption with Cl^–^ on the Ag surface. However, due to the poor solubility of AgCl in acetone, Cl^–^ cannot substantially desorb from the Ag surface. As a result, when the particles are collected by centrifugation and then redispersed in water, the PVP loops become less collapsed, allowing the Ag nanocubes to be well dispersed. Simultaneously, as a polar solvent, water can exert a strong solvation effect on AgCl, promoting the desorption of Cl^–^ from the surface and the adsorption of more carbonyl groups from PVP onto the surface as acetone is largely removed during the washing process.

**Figure 4:**
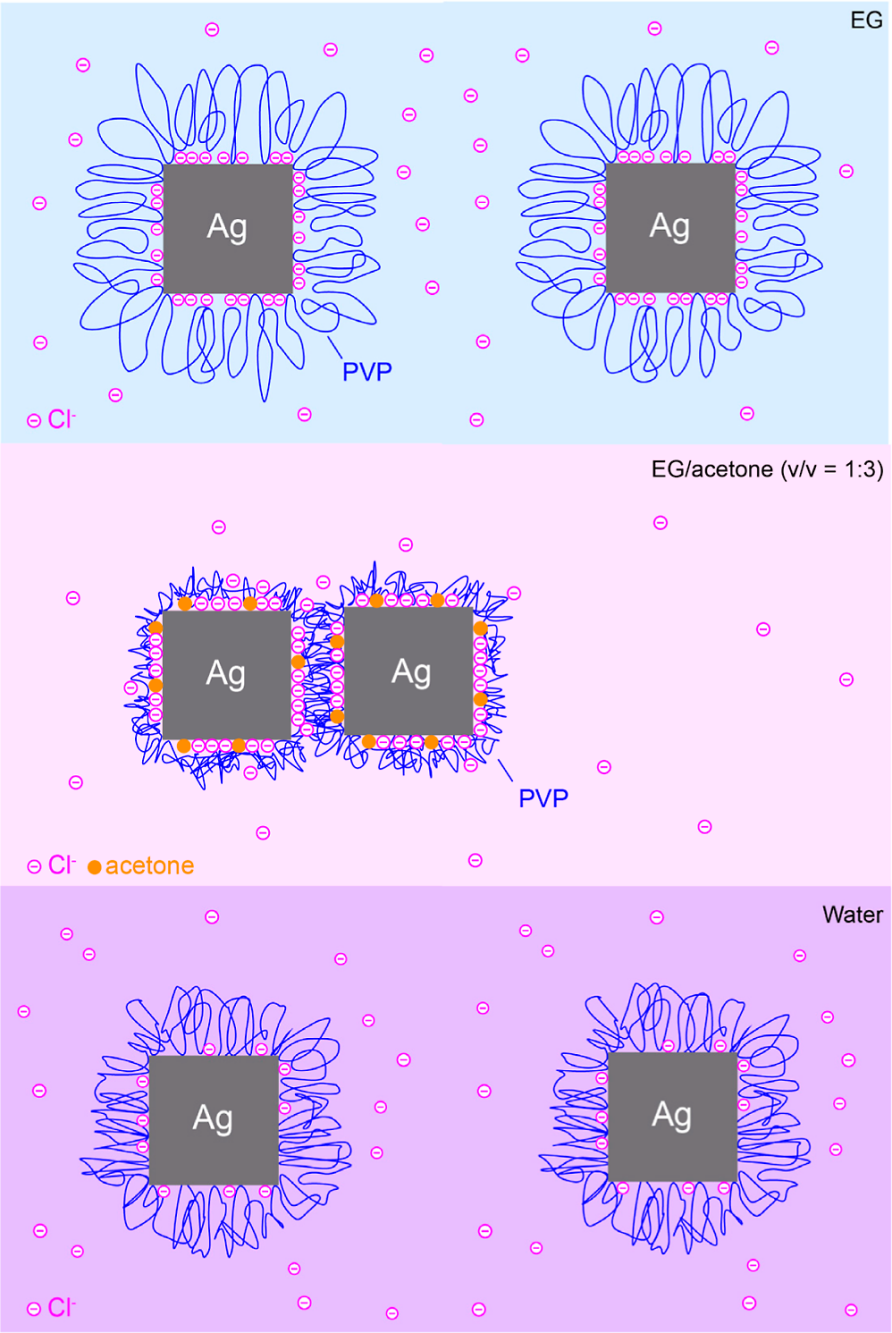
A model detailing the surface of two Ag nanocubes suspended in the original reaction solution (top panel), after dilution with acetone (middle), and after collection by centrifugation and then redispersion in water (bottom).

To better understand how different solvents affect the surface of Ag nanocubes, we performed SERS measurements on the reaction solution after it had been diluted by three times in volume with EG, water, ethanol, and acetone, respectively. We first focused on the changes to ν_Ag–Cl_ in different solvents. As shown in Figure 5, for the samples diluted with EG and water, the area of the ν_Ag–Cl_ peak decreased proportionally with the dilution factor. The stronger ν_Ag–Cl_ peak for the sample diluted with ethanol relative to that diluted with water indicates more AgCl was deposited on the surface of the nanocube when suspended in the EG–ethanol mixture than in the EG–water mixture, likely due to the difference in AgCl solubility. As discussed above, the significant enhancement of SERS signal in samples diluted with acetone was caused by particle aggregation. The ν_Ag–Cl_ peak only shifted when diluted with acetone, indicating that the coverage density of Cl^–^ on the Ag surface had changed, whereas no shift was observed upon the addition of water or ethanol. Meanwhile, a weak peak was observed around 1762 cm^–1^ for the samples diluted with water and ethanol, indicating more carbonyl groups from PVP anchored to or in proximity to the surface. Based on these observations, we proposed that acetone promoted competitive adsorption between carbonyl groups and Cl^–^ on the Ag surface, increasing the coverage density of carbonyl groups relative to the Ag nanocubes suspended in the original reaction solution. In contrast, water and ethanol primarily promoted the adsorption of carbonyl groups of PVP at pre-existing surface sites unoccupied by Cl^–^. Both situations significantly retarded the adsorption of O_2_ on the surface of Ag nanocubes, protecting the sharp corners and edges from truncation via oxidative etching.

**Figure 5:**
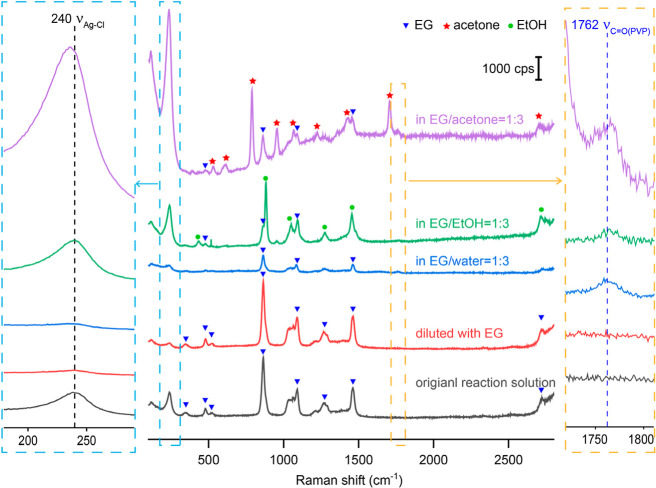
SERS spectra of Ag nanocubes suspended in the original reaction solution or diluted by a factor of 4 in volume with EG, water, acetone, and ethanol, respectively. The sample was allowed to sit for 40 min prior to SERS measurement.

## Conclusions

In summary, we have used SERS to analyze and elucidate changes to the surface of Ag nanocubes during sample preparation after their HCl-mediated polyol synthesis. Our results revealed the coadsorption between Cl^–^ ions and the carbonyl group from acetone added during the collection step, helping suppress oxidation etching. Meanwhile, diluting the original reaction mixture with more than two volumes of acetone also induced reversible particle aggregation, greatly improving the sample collection efficiency. Washing the collected particles with water allowed the nanocubes to be redispersed by promoting the desorption of Cl^–^ ions and the adsorption of the carbonyl group from PVP. To further understand solvent effect, we directly diluted the reaction solution by the same factor with EG, water, acetone, and ethanol, and collected the corresponding SERS spectra. The redshift in the ν_Ag–Cl_ peak could only be observed in the sample diluted with acetone, while the carbonyl signal from PVP could only be observed in the samples diluted with water and EtOH. Again, these data suggested that the addition of acetone into the original reaction solution resulted in coadsorption of its carbonyl group with Cl^–^ ions, whereas the addition of water and ethanol facilitated the adsorption of the carbonyl group from PVP. Collectively, these findings suggest that the seemingly routine steps of sample collection, washing, and redispersion can significantly affect the surface chemistry and even morphology of Ag nanocubes, with direct consequences on their properties and applications. The insights from this study can provide guidance for optimizing protocols related to the preparation and preservation of Ag nanocubes, thereby improving their reproducibility and stability.

## Supplementary Material



## Notes

TEM images of the as-prepared Ag nanocubes; TEM images and UV–vis spectra of the Ag nanocubes after storage at room temperature and under ambient conditions in the original reaction solution without/with dilution with different solvents (PDF)
