# Supplementary material for: Seeing Is Believing: How Does the Surface of Silver Nanocubes from a Polyol Synthesis Change during Sample Collection, Washing, and Redispersion
Source: Langmuir. 2025 Jul 22;41(30):20272–9. doi: 10.1021/acs.langmuir.5c02610 (PMC12333423; doi:10.1021/acs.langmuir.5c02610)
Supplement: Supplementary file 1 [file la5c02610_si_001.pdf]

Supporting Information

Seeing Is Believing: How Does the Surface of Silver Nanocubes from a Polyol Synthesis Change during Sample Collection, Washing, and Redispersion

Qijia Huang,[a] and Younan Xia[ab]*

[a]School of Chemistry and Biochemistry, Georgia Institute of Technology, Atlanta, Georgia 30332, United States

[b]The Wallace H. Coulter Department of Biomedical Engineering, Georgia Institute of Technology and Emory University, Atlanta, Georgia 30332, United States

*Corresponding authors. E-mails: younan.xia@bme.gatech.edu

Number of pages: 4
Number of figures: 3

Table of Contents

Figure S1. TEM image of the as-prepared Ag nanocubes.
Figure S2. TEM images of Ag nanocubes stored in reaction solution without/with dilution with different solvents.
Figure S3. UV-vis spectra of Ag nanocubes stored in reaction solution without/with dilution with different solvents.

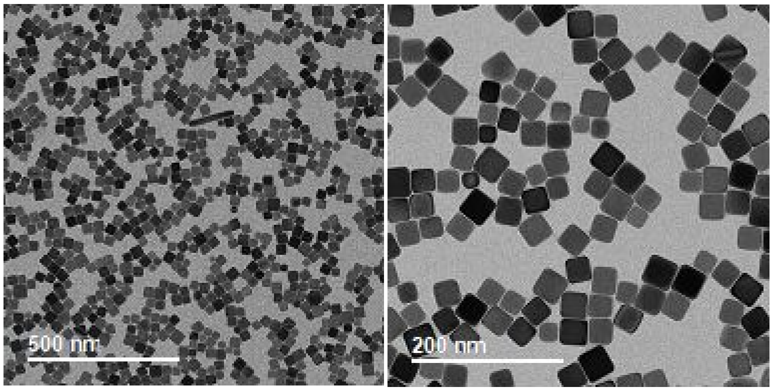

Figure S1. TEM image of the as-prepared Ag nanocubes. After synthesis, the reaction mixture was immediately cooled in an ice bath, and then collected by centrifugation after adding acetone, washed twice with water, and directly redispersed in water for TEM sample preparation.

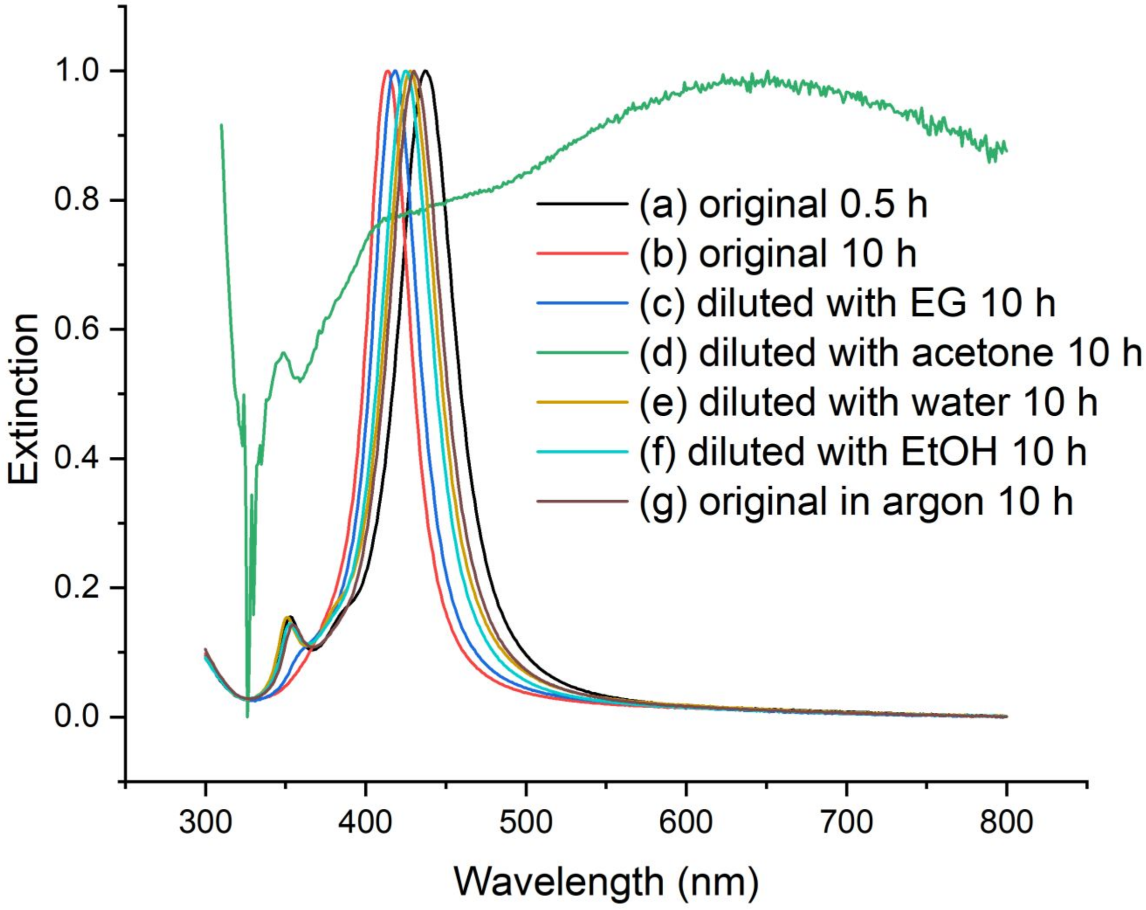

Figure S2. UV-vis spectra of Ag nanocubes original reaction solution, which was quenched with an ice bath, followed by: (a) storage in air for 0.5 hours, (b) storage in air for 10 hours, (c) diluted with EG and storage in air for 10 hours, (d) diluted with acetone and storage in air for 10 hours, (e) diluted with water and storage in air for 10 hours, (f) diluted with EtOH and storage in air for 10 hours, (g) protected with argon for 10 hours. The dilution factor for c–f is four.

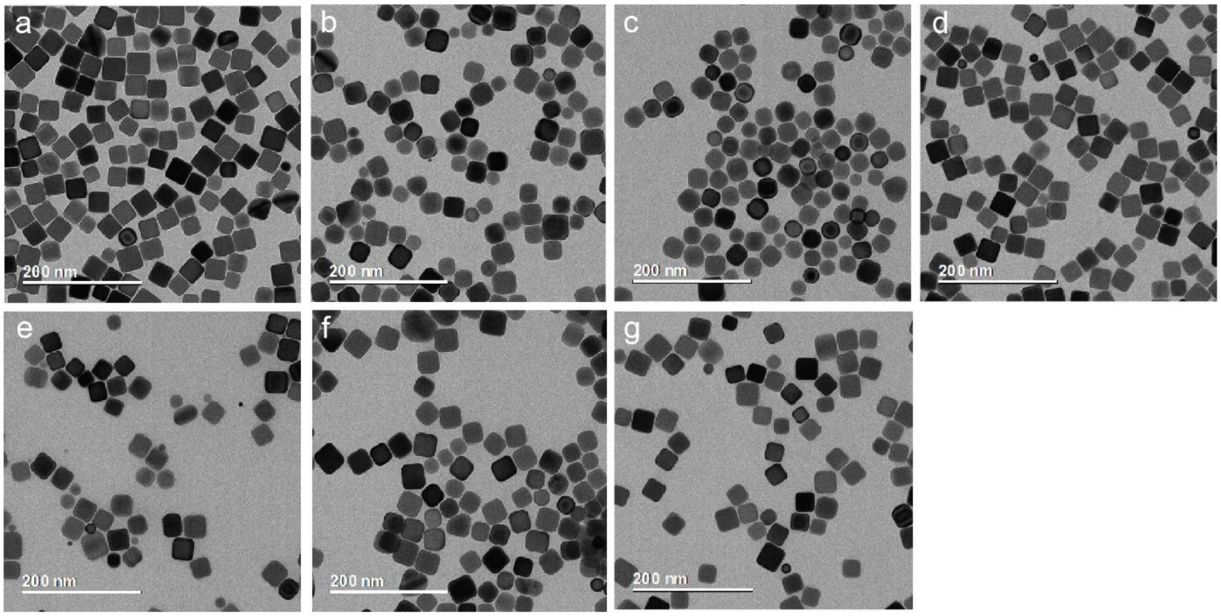

Figure S3. TEM images of Ag nanocubes in their original reaction solution, which was quenched with an ice bath, followed by: (a) storage in air for 0.5 hours, (b) storage in air for 10 hours, (c) diluted with EG and storage in air for 10 hours, (d) diluted with acetone and storage in air for 10 hours, (e) diluted with water and storage in air for 10 hours, (f) diluted with EtOH and storage in air for 10 hours, (g) protected with argon for 10 hours. The dilution factor for c–f is four.
